# Explainable Machine Learning Models for Predicting Functional and Pain Recovery After Fragility Fracture Surgery

**DOI:** 10.3390/diagnostics16172875

**Published:** 2026-09-07

**Authors:** Chien-Hung Chen, Chin-Kai Huang, Chih-Cheng Lai, Li-Chuan Lin, Tai-Hua Yang

**Affiliations:** 1 Department of Biomedical Engineering, National Cheng Kung University (NCKU), No. 1, University Road, Tainan 701, Taiwan; 2 Department of Orthopaedic Surgery, Kaohsiung Veterans General Hospital Tainan Branch, Tainan 710, Taiwan; 3 Department of Intensive Care Medicine, Chi Mei Medical Center, Tainan 901, Taiwan; 4 Institute of Physical Education, Health and Leisure Studies, National Cheng Kung University, Tainan 701, Taiwan; 5 Department of Orthopaedic Surgery, National Cheng Kung University Hospital, College of Medicine, National Cheng Kung University, Tainan 701, Taiwan; 6 Center for Biomaterials Research, National Cheng Kung University, Tainan 701, Taiwan; 7 Medical Device Innovation Center, National Cheng Kung University, Tainan 701, Taiwan

**Keywords:** fragility fracture surgery, machine learning, pain, prediction, recovery

## Abstract

**Background:** Recovery after hip fragility fracture surgery is heterogeneous. Explainable machine learning (ML) may help identify factors associated with short-term recovery during inpatient rehabilitation. **Methods:** This retrospective exploratory cohort included 88 patients who underwent surgery at our institution or two outside hospitals and received inpatient rehabilitation at our institution between 2017 and 2019. Improvements at discharge in activities of daily living (ADL), Harris Hip Score (HHS), and numeric rating scale (NRS) pain were defined using clinically established or data-derived thresholds. Admission demographic, clinical, and laboratory variables were analyzed using logistic regression, random forest, and XGBoost with fixed model configurations and class weighting. Model performance was assessed as a secondary feasibility analysis using repeated stratified 5-fold cross-validation (50 splits), corrected resampled *t*-tests, DeLong’s tests, and McNemar’s tests with Bonferroni correction. TreeSHAP was applied to out-of-fold predictions, and feature stability was evaluated across folds. **Results:** Discrimination was modest (AUC, 0.52–0.67), without significant between-model differences after correction. Prominent features were blood urea nitrogen, age, hemoglobin, and sodium for ADL; ALT (GPT), cardiovascular comorbidity, blood urea nitrogen, and age for HHS; and DXA, platelet count, sodium, and age for NRS. Stability analyses supported recurring feature associations across cross-validation folds. **Conclusions:** Explainable ML may be feasible as a hypothesis-generating approach for exploring factors associated with short-term recovery after fragility fracture surgery.

## 1. Introduction

Fragility fractures, most commonly affecting the hip and spine, represent a growing public health challenge in aging populations [1]. Globally, approximately 8.9 million osteoporotic fractures occur each year [2]. Hip fractures alone affect over 1.6 million individuals annually [3], a number expected to rise dramatically to more than 6 million by 2050 as the global population ages [4]. In East Asia, the burden is increasing rapidly, with incidence rates among older adults ranging between 150 and 250 per 100,000 people [5]. These injuries carry profound consequences, including prolonged hospitalization, loss of independence, and increased mortality. Studies show that 20–30% of patients die within a year of a hip fracture, and fewer than half regain their pre-fracture functional status [6]. The associated healthcare costs and societal burden are likewise substantial.

Despite advances in surgical techniques, anesthesia, and perioperative care, recovery following fragility fracture surgery remains highly variable. Many patients continue to experience long-term disability, reduced independence in activities of daily living (ADL), and persistent pain, even after technically successful procedures [7]. Accordingly, early identification of patients at risk for poor postoperative outcomes remains an important clinical goal. Prognostic information may support clinical decision-making by informing individualized rehabilitation planning, improving resource allocation, and facilitating shared decision-making between patients and providers [8].

Predicting patient outcomes has traditionally relied on statistical models that incorporate demographic characteristics, comorbidities, and intraoperative variables. While these approaches offer valuable insights, they often assume linear relationships and may struggle to capture the complex, multi-factorial nature of surgical recovery [9]. In contrast, machine learning (ML) techniques are well suited to exploring high-dimensional clinical data and modeling non-linear interactions among diverse variables [10]. However, the clinical applicability of ML-based approaches remains limited by concerns regarding interpretability, robustness, and generalizability, particularly in small or heterogeneous cohorts [11].

Explainable artificial intelligence (XAI) methods have emerged as a promising approach to improving model interpretability. Shapley Additive Explanations (SHAP) provide intuitive and quantitative insights into the relative contribution of individual features to model outputs [12]. In the context of fragility fracture recovery, explainable ML frameworks may help identify clinical factors associated with functional and pain improvement, thereby offering insights that could inform hypothesis generation and future targeted interventions.

The primary objective of this study was to identify key clinical factors associated with functional and pain recovery by applying an explainable ML framework. Secondarily, we aimed to evaluate the feasibility of using these models to capture non-linear relationships in a fragility fracture cohort, serving as an exploratory pilot for future large-scale predictive modeling.

## 2. Methods

### 2.1. Data Source and Participants

We retrospectively reviewed the medical records of patients who underwent hip fragility fracture surgery either at our institution—the Tainan Branch of Kaohsiung Veterans General Hospital, a district hospital—or at one of two outside hospitals: a tertiary medical center (National Cheng Kung University Hospital [NCKUH]) and a regional hospital (Tainan Hospital, Ministry of Health and Welfare [Tainan Hospital, MOHW]). Patients who underwent surgery at the outside hospitals were subsequently referred to our institution for postoperative inpatient rehabilitation between November 2017 and April 2019.

Patients with missing essential hospitalization records or incomplete functional outcome assessments (i.e., missing either the pre- or post-rehabilitation assessment) were excluded, resulting in an eligible cohort of 91 patients.

During data preprocessing, two patients with physiologically implausible laboratory values (DXA = −48.0 and RDW = 6.8%), suggesting data entry errors, and one patient with all hematology and biochemistry variables missing were further excluded because laboratory-based predictors could not be calculated. The final analytic sample comprised 88 patients.

### 2.2. Ethics Statement

The study protocol was reviewed and approved by the Institutional Review Board (IRB) of Kaohsiung Veterans General Hospital (IRB No. KSVGH26-CT3-18). All research procedures adhered to the ethical standards outlined in the Declaration of Helsinki and followed the guidelines of the International Council for Harmonization on Good Clinical Practice (ICH-GCP). Given the retrospective nature of the study and the use of anonymized data, the IRB granted a waiver of informed consent.

### 2.3. Definition of Functional Gains and Pain Reduction

Functional and pain outcomes—including activities of daily living assessed by the Barthel Index (BI; range, 0–100, where higher scores indicate greater independence) [13], hip function evaluated by the Harris Hip Score (HHS; range, 0–100, incorporating pain, function, deformity, and range of motion) [14], and pain intensity measured by the Numeric Rating Scale (NRS; range, 0–10)—were assessed upon admission to the inpatient rehabilitation ward (on the admission day or the preceding day) and reassessed at discharge (on the discharge day or the preceding day).

Treatment outcome (improvement) for each measure was defined as the change between the baseline (admission) and discharge assessments, reflecting functional gain over the course of rehabilitation rather than the entire post-surgical period. For binary modeling, patients reaching the predefined target threshold for each outcome measure were classified as the ‘Improved’ group, whereas those who did not meet the criteria were assigned to the ‘Not-improved’ group. Accordingly, the evaluation interval corresponded to the duration of inpatient rehabilitation, with a mean length of stay of 13.6±3.1 days (median, 14 days; range, 2–22 days; data unavailable for four patients). Because the present study focused on the relationship between patients’ pre-admission physiological status and functional recovery, length of stay was excluded as a model predictor, as it reflects the rehabilitation process rather than a baseline characteristic. Consequently, missing length-of-stay data did not affect the final analytic sample.

To define binary targets for supervised machine learning, we evaluated minimal important change (MIC) thresholds reported in a comparable post-acute hip fracture cohort. However, these MIC values were estimated using distribution-based methods (half standard deviation), reflecting statistical variation rather than patient-perceived meaningful improvement [15]. Furthermore, applying these low thresholds would classify the vast majority of our cohort as responders, creating severe class imbalance. Therefore, we operationalized outcome targets using literature-informed thresholds for moderate-to-substantial improvement, defined as follows:

For HHS, clinically meaningful improvement was defined as a score gain of 40 points or greater (HHS change score of 40 or more), corresponding to the established threshold for moderate improvement (39.6–40.1 points) reported in primary total hip arthroplasty cohorts [16].

For the Barthel Index (BI), given the lack of a validated MIC or clinically established threshold for moderate improvement in the Barthel Index (BI) among older adults in post-acute hip fracture rehabilitation, we operationalized a change threshold of an increase of ≥25 points. This threshold is empirically grounded in findings from post-acute care and geriatric rehabilitation cohorts, where mean functional gains were reported to be 23.7 points during PAC [15] to 24.6 points at 3 months post-discharge [17].

For pain recovery, a primary threshold of an NRS score reduction of ≥3 points was operationalized. Given our cohort’s mean baseline NRS score of 6.11 points (SD 2.40), a 3-point reduction corresponds to an approximately 50% decrease in pain intensity, aligning with the IMMPACT consensus definition for substantial clinically important pain relief [18].

### 2.4. Data Collection and Feature Engineering

The clinical dataset included 88 patients with demographic characteristics (gender, age, hospital-related items), fracture characteristics (type, side, location, and surgical category), along with functional scores assessed at rehabilitation ward admission (ADL1, NRS1, HHS1) and at discharge (ADL2, NRS2, HHS2), score changes, and binary indicators of improvement. Additional variables comprised comorbidities (i.e., diabetes, hypertension, coronary artery disease, cerebrovascular disease, malignancy, neurological, cardiovascular, renal, genitourinary, and liver/endocrine disease), bone mineral density by DXA, and laboratory data obtained at rehabilitation ward admission. Comorbidities were identified using International Classification of Diseases codes, as listed in Supplementary Table S1. Laboratory measures included complete blood count (i.e., white blood cell count, red blood cell count, hemoglobin, hematocrit, mean corpuscular volume, mean corpuscular hemoglobin, mean corpuscular hemoglobin concentration, and red blood cell distribution width), platelet count, liver and renal function tests (i.e., liver enzymes, Serum Glutamic Oxaloacetic Transaminase [SGOT] and Serum Glutamic Pyruvic Transaminase [SGPT]; and for kidney function, Blood urea nitrogen [BUN] and Glomerular Filtration Rate [GFR]); and serum electrolytes (i.e., sodium, and potassium).

To address multicollinearity identified via variance inflation factor (VIF/GVIF) analysis, several correlated variables were excluded prior to model development. Given the high VIF observed among several hematologic indices, only hemoglobin and red blood cell distribution width were retained to represent anemia-related status, as hemoglobin serves as the clinical gold standard for anemia assessment, while red blood cell distribution width is an independent predictor of frailty and all-cause mortality in older adults. Similarly, creatinine was excluded in favor of estimated GFR, and coronary artery disease was subsumed within the broader cardiovascular disease category due to substantial overlap. Liver/endocrine comorbidity was also excluded owing to its high collinearity with DXA and its low prevalence (n = 1) in this cohort. Length of stay (LOS) was similarly excluded for the reasons outlined in Section 2.3, and hospital source (admission origin) was excluded following clinical review, as it was judged to primarily reflect administrative referral patterns rather than a baseline physiological characteristic. The final model, therefore, included 8 comorbidity categories, DXA, hemoglobin, RDW, platelet count, and 6 biochemistry variables (GOT, GPT, BUN, sodium, potassium, and GFR), in addition to gender, age, and surgical category.

Data preprocessing was tailored to the requirements of the machine learning algorithms employed. To minimize the risk of label leakage, discharge-time functional scores (ADL2, NRS2, HHS2) and outcome-related delta variables (e.g., ADL diff, NRS diff, HHS diff, and their corresponding binary improvement indicators) were excluded from the predictor set. Baseline functional scores obtained at rehabilitation ward admission (ADL1, NRS1, HHS1) were also excluded, as this study focused on identifying physiological, demographic, and comorbidity-based predictors of recovery rather than using baseline functional status itself as a predictor. Gender and surgical category were encoded as binary (0/1) indicator variables, while continuous variables were retained in their original scales without normalization. Missing values in baseline predictor variables were imputed using the median of each feature: for descriptive statistics and the multicollinearity assessment (VIF/GVIF), imputation used the median computed from the complete sample; for model training, cross-validation, and the out-of-fold SHAP analysis (Section 2.7), imputation was instead performed within a pipeline embedded inside each cross-validation fold, computing the median exclusively from the training-fold data and applying it to both folds via transformation, to prevent validation-set information from influencing the imputed values.

### 2.5. Model Development

We implemented three supervised machine learning algorithms—logistic regression (LR), random forest (RF), and extreme gradient boosting (XGBoost)—to classify patients achieving clinically meaningful rehabilitation improvements across functional and pain outcomes. The proportion of improved versus non-improved patients differed across outcomes—most notably for HHS, where only 23 of 88 patients reached the 40-point improvement threshold—so class imbalance was addressed for every outcome using built-in class weighting rather than synthetic oversampling, so that the feature contributions estimated by SHAP would reflect the original clinical distribution rather than artificially generated samples.

For LR and RF, the class_weight parameter was set to balance the influence of each outcome category automatically; for XGBoost, a scale_pos_weight proportional to the inverse class frequency was applied. We applied the same imbalance-handling strategy and the same candidate hyperparameters uniformly across ADL, HHS, and NRS, rather than tuning each outcome separately, so that the three outcomes could be compared on equal footing.

Because the sample size was small (n = 88), to reduce the risk of overfitting, we pre-specified a strategy to compare a conservative, regularized configuration for each algorithm—logistic regression with a stronger L2 penalty (C = 0.1), random forest with a larger minimum leaf size (min_samples_leaf = 5), and XGBoost with a larger minimum child weight (min_child_weight = 5)—against its corresponding default settings using a corrected resampled *t*-test on specificity and balanced accuracy.

To avoid outcome-specific overfitting and maintain consistency across tasks, our pre-specified plan dictated that unless a regularized configuration yielded statistically significant improvements over default, hyperparameter selection would be guided by algorithm-specific overfitting risks in small samples: specifically, retaining RF in its regularized form (min_samples_leaf = 5) due to its high susceptibility to overfitting when the sample size is small, while keeping LR and XGBoost at their default settings. This resulting single hybrid configuration was applied identically across all three outcomes (ADL, HHS, and NRS) for subsequent model evaluations.

### 2.6. Model Evaluation

Model discrimination and classification performance were evaluated using five standard metrics: area under the receiver operating characteristic curve (AUC), balanced accuracy, sensitivity, specificity, and F1 score. All metrics were computed across the 50 splits of the repeated stratified 5-fold cross-validation scheme. Given the modest sample size (n = 88), these indicators are presented as descriptive, feasibility-level performance metrics rather than definitive validated estimates.

To guide hyperparameter selection, we first performed a sensitivity analysis comparing the default and regularized configurations of each algorithm. Specifically, a corrected resampled *t*-test was applied to specificity and balanced accuracy across the 50 splits. These two metrics were prioritized over AUC because they are more sensitive to majority-class overfitting under severe class imbalance—the key failure mode regularization was designed to mitigate.

Following the specification of the final hybrid configurations, we compared the three algorithms within each outcome using three complementary statistical tests:

1. Corrected resampled *t*-test: Applied to AUC values across all 50 repeated splits, incorporating a variance correction factor to explicitly account for the correlation structure introduced by overlapping folds.
2. DeLong’s test: Applied to out-of-fold ROC curves from a single stratified 5-fold split to evaluate paired AUC differences.
3. McNemar’s test: Applied to the corresponding out-of-fold binary predictions (at a 0.5 threshold) from the same single 5-fold split to compare classification agreement.

DeLong’s and McNemar’s tests were evaluated on a single 5-fold split because both require paired predictions on an identical, non-overlapping patient sample to satisfy their independence assumptions, which repeated overlapping folds would violate. All pairwise comparisons were adjusted for multiple testing using Bonferroni correction (α = 0.05/9 = 0.0056).

### 2.7. Model Interpretation

As the primary interpretability analysis, feature contributions were evaluated using TreeSHAP applied to the primary tree-based ensemble models (Random Forest and XGBoost) for each clinical outcome. To mitigate optimistic bias inherent in explaining models on training data, SHAP values were calculated out-of-fold (OOF): for each of the 50 splits generated by the repeated stratified 5-fold cross-validation (10 repeats), TreeSHAP was applied exclusively to held-out validation patients using that fold’s fitted model. Each patient’s SHAP values were averaged across the 10 folds in which they were held out to derive a robust, patient-level OOF SHAP representation.

These OOF SHAP values were utilized to construct summary beeswarm plots and calculate global mean absolute SHAP values ($\mathrm{mean}\left(\left|\mathrm{SHAP}\;\mathrm{value}\right|\right)$), characterizing both the directionality and quantitative magnitude of each feature’s predictive contribution.

Furthermore, to evaluate feature consistency across resampling iterations, per-fold SHAP values were used to perform a rank stability audit across all 50 folds; the mean rank, standard deviation of rank, and the frequency of appearing within the top 5 predictors (Top-5 Rate %) were quantified. Finally, for top-ranking continuous features demonstrating high stability, TreeSHAP dependence plots were constructed to characterize potential non-linear relationships and clinically relevant inflection thresholds.

## 3. Results

### 3.1. Patients’ Characteristics and Functional Outcomes

We first summarize the raw ADL, HHS, and NRS scores at rehabilitation ward admission and discharge, along with their corresponding score changes. ADL scores increased from a mean of 47.10 (SD 6.94) at admission to 71.31 (SD 13.44) at discharge, corresponding to a mean change of 24.20 (SD 11.79; median 25.00, min −5.00, max 50.00). HHSs increased from a mean of 34.81 (SD 8.33) at admission to 66.35 (SD 12.08) at discharge, a mean change of 31.55 (SD 11.42; median 33.00, min 6.00, max 56.00). NRS pain scores decreased from a mean of 6.11 (SD 2.40) at admission to 2.48 (SD 1.83) at discharge, a mean change of −3.64 (SD 1.99; median −3.50, min −10.00, max 0.00).

Based on the outcome-specific thresholds (Section 2.3), patients were categorized as improved or not improved. Overall, 52.3% achieved improvement in ADL (46/88), 26.1% in HHS (23/88), and 64.8% in NRS (57/88).

Baseline characteristics of the 88 patients by improvement status for each outcome are summarized in Table 1. This table is intended to describe the distribution of baseline characteristics between the two groups for each outcome; given the large number of variables shown, no formal hypothesis testing was performed to avoid inflating the risk of false-positive findings from multiple univariate comparisons, and the table should be read descriptively rather than as confirmatory group comparisons. For ADL, the improved group was numerically younger and had lower BUN and higher sodium than the non-improved group. For HHS, the improved group was numerically younger, included a higher proportion of females, and had a markedly higher prevalence of cardiovascular comorbidity, lower BUN, and higher platelet count and eGFR than the non-improved group; because the improved HHS group was small (n = 23), these baseline patterns should be interpreted cautiously. For NRS, the improved group had numerically lower bone mineral density (more negative DXA T-score), higher sodium, higher eGFR, and lower BUN than the non-improved group. No large differences in comorbidity burden other than cardiovascular disease (for HHS) were observed between groups.

### 3.2. Sensitivity Analysis and Hybrid Model Performance

As pre-specified in Section 2.5, we evaluated whether stronger regularization improved performance over default configurations in our small sample size (n = 88). Across all three outcomes (ADL, HHS, and NRS), the corrected resampled *t*-test revealed no statistically significant differences between the regularized and default configurations for any model in either specificity or balanced accuracy (all *p* > 0.05; Supplementary Table S2). Overall, stronger regularization yielded only minor numerical variations without altering the substantive findings.

Specifically, for ADL, stronger regularization led to slight, non-significant AUC improvements across all three models (0.671 → 0.693 for LR; 0.599 → 0.601 for RF; 0.624 → 0.635 for XGBoost). For HHS—the outcome with the most severe class imbalance (23/88 responders)—regularization resulted in slight AUC declines for LR (0.601 → 0.562) and XGBoost (0.636 → 0.610), whereas RF exhibited modest gains in both AUC (0.651 → 0.668) and balanced accuracy (0.580 → 0.606). For NRS, LR and XGBoost showed minor numerical improvements under regularization, while RF showed a slight decline in AUC (0.587 → 0.566).

Because the sensitivity analysis revealed no statistically significant differences between default and regularized configurations for any algorithm, hyperparameter selection was guided by algorithm-specific overfitting risks in small samples rather than empirical, outcome-specific tuning. Given that unconstrained random forests are particularly prone to overfitting when the sample size is small (n = 88), we retained RF in its regularized form (min_samples_leaf = 5), whereas LR and XGBoost were kept at their default settings.

**Figure 1:**
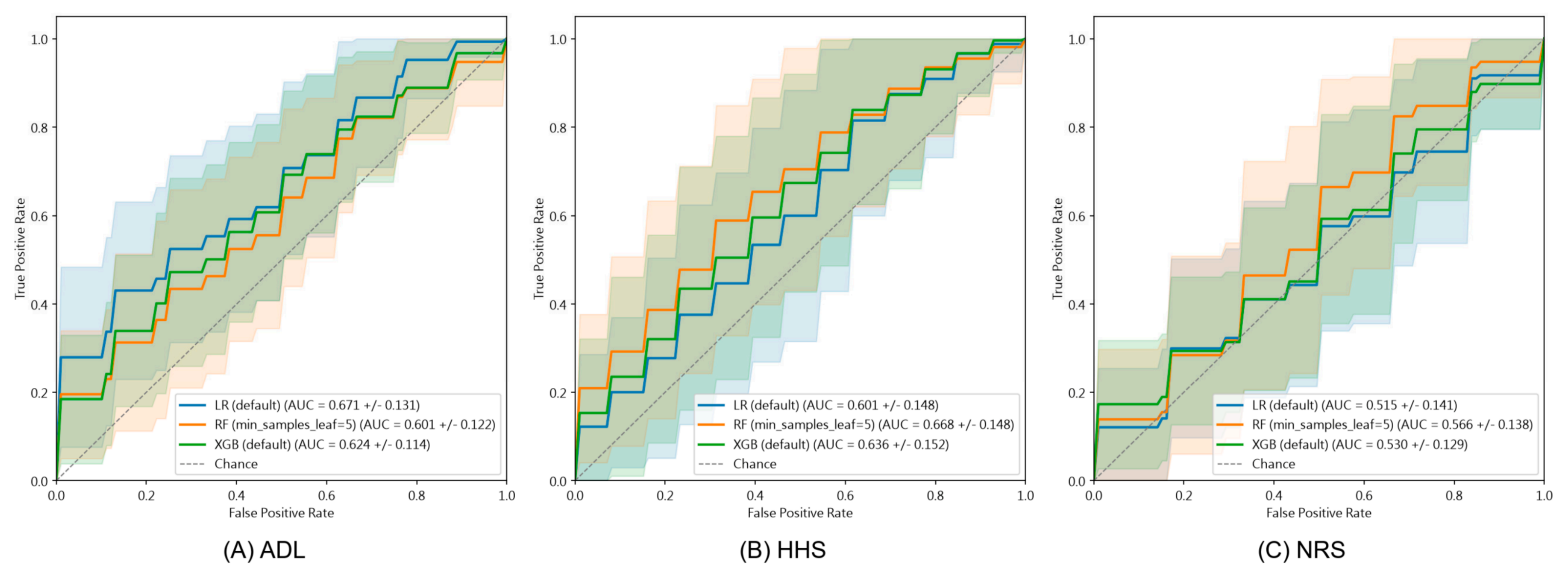
Receiver operating characteristic (ROC) curves of the hybrid model configurations across 50 splits of repeated stratified 5-fold cross-validation. Panels depict model discrimination for (**A**) ADL, (**B**) HHS, and (**C**) NRS improvement thresholds. Solid lines represent the mean ROC curves across 50 splits, and shaded regions denote ±1 standard deviation (SD). LR, logistic regression (default); RF, random forest (min_samples_leaf = 5); XGB, Extreme Gradient Boosting (default); Chance, random performance baseline (AUC = 0.50).

Consequently, all subsequent analyses utilized this pre-specified hybrid model configuration (LR default, RF with min_samples_leaf = 5, XGB default). Table 2 summarizes the predictive performance of this hybrid configuration across ADL, HHS, and NRS improvement using repeated stratified 5-fold cross-validation (50 splits; mean ± SD). Discrimination performance across all three outcomes is further depicted in Figure 1.

Overall, model performance across all three functional outcomes reflected a weak-to-modest predictive signal. In predicting ADL improvement, discrimination was modest, with AUC ranging from 0.601 (RF) to 0.671 (LR), F1 scores ranging from 0.591 to 0.614, and balanced accuracy ranging from 0.565 to 0.604. For HHS improvement—the outcome with the most severe class imbalance (n = 23 responders)—AUC ranged from 0.601 (LR) to 0.668 (RF), and F1 scores ranged from 0.310 to 0.420, with RF achieving numerically higher performance on both metrics (AUC 0.668, F1 0.420). For NRS pain relief, discrimination was low across all three models (AUC 0.515–0.566; balanced accuracy 0.502–0.560), reflecting the inherent challenge of predicting pain reduction at discharge solely from baseline features upon admission to the rehabilitation unit.

Pairwise performance comparisons among LR, RF, and XGBoost were evaluated using three complementary statistical tests: a corrected resampled *t*-test on cross-validation AUC values, DeLong’s test on out-of-fold ROC curves from a single stratified 5-fold split, and McNemar’s test on out-of-fold binary classification outcomes at the 0.5 decision threshold. After Bonferroni correction for multiple comparisons, none of the pairwise comparisons achieved statistical significance for any outcome across any of the three tests. While uncorrected McNemar tests suggested nominal differences between LR and XGBoost for both HHS (*p* = 0.009) and NRS (*p* = 0.025), these effects did not remain significant following correction.

Furthermore, while point estimates for ADL and HHS were numerically higher than those for NRS, these variations should be interpreted cautiously due to wide confidence intervals and the small number of responders in HHS (n = 23), which limits the reliability of individual point estimates. Consequently, predictive performance across all three outcomes is best characterized as exploratory.

### 3.3. SHAP Feature Importance and Dependence Analysis

#### 3.3.1. Global Feature Importance and Stability Audits

**Figure 2:**
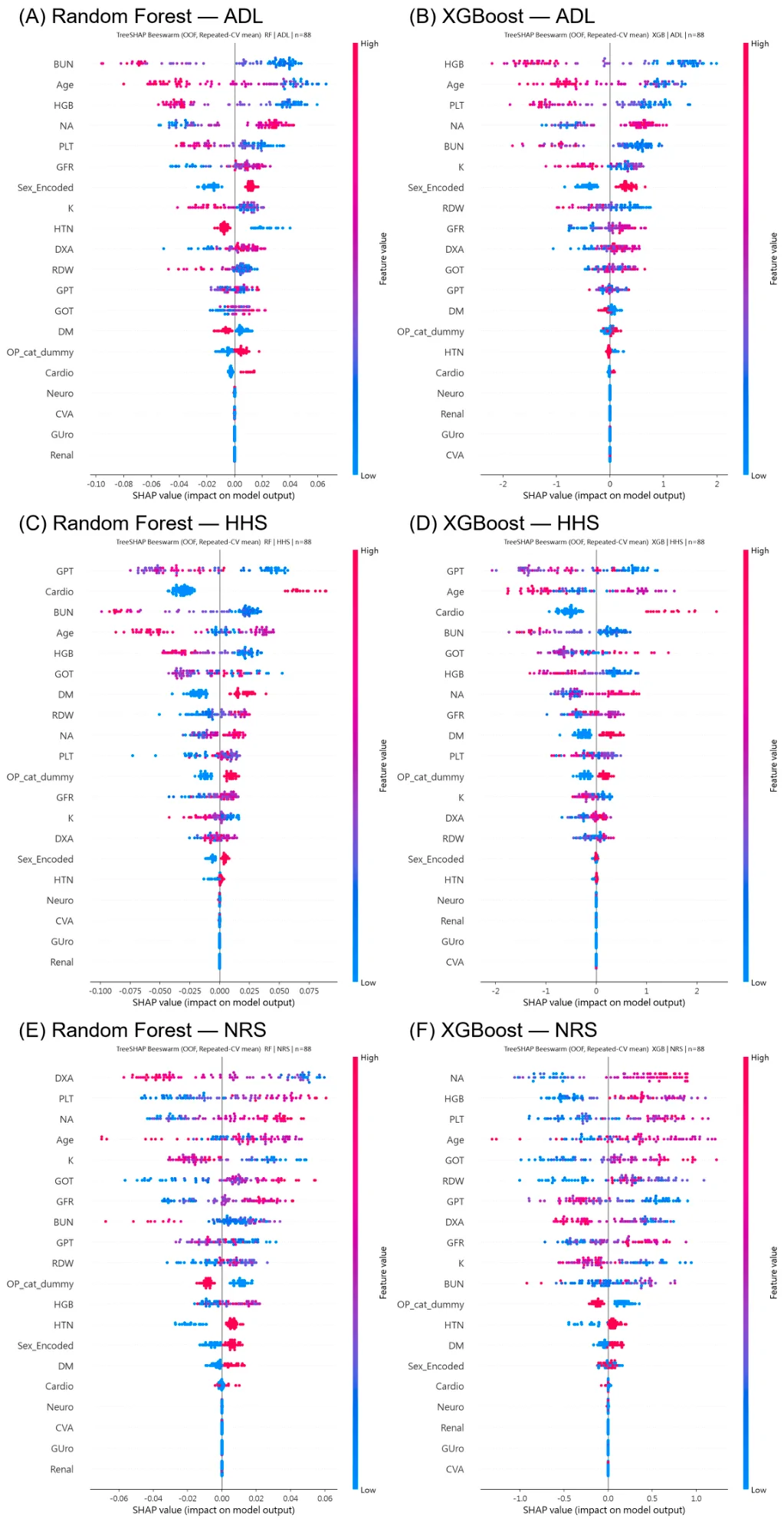
Out−of-fold TreeSHAP summary beeswarm plots illustrating feature contributions to functional and pain outcomes. Panels depict global feature contributions comparing Random Forest (left column: (**A**,**C**,**E**)) and XGBoost (right column: (**B**,**D**,**F**)) across three clinical outcomes: ADL improvement (**A**,**B**), HHS improvement (**C**,**D**), and NRS pain relief (**E**,**F**). Each dot represents an individual patient (n = 88), with color denoting feature magnitude (red = high, blue = low) and horizontal displacement showing prediction impact (positive values indicate a higher likelihood of improvement). ADL, activities of daily living; BUN, blood urea nitrogen; Cardio, cardiovascular comorbidity; DXA, dual-energy X-ray absorptiometry T-score; GFR, glomerular filtration rate; GOT, glutamic oxaloacetic transaminase (AST); GPT, glutamic pyruvic transaminase (ALT); HGB, hemoglobin; HHS, Harris Hip Score; NRS, Numeric Rating Scale; PLT, platelet count; RDW, red cell distribution width; SHAP, Shapley additive explanations.

TreeSHAP beeswarm analysis (Figure 2) demonstrated consistent feature contributions across outcomes in both tree-based models. For ADL improvement (Figure 2A,B), hemoglobin, blood urea nitrogen (BUN), age, and sodium were the primary stable predictors across 50 cross-validation folds, highlighting the pivotal roles of baseline hematologic status and electrolyte balance in functional recovery. For HHS improvement (Figure 2C,D), ALT (GPT), cardiovascular comorbidity, BUN, and age emerged as key shared drivers. For NRS pain relief (Figure 2E,F), bone mineral density (DXA), platelet count, sodium, and age consistently anchored model predictions, emphasizing skeletal, hematologic, and electrolyte status as core biological correlates of pain recovery.

Global feature importance magnitudes (Mean ∣SHAP∣) cross-validation rank stability audits confirmed strong predictor convergence between algorithms (Table 3 and Supplementary Table S3).

For ADL improvement, hemoglobin and age demonstrated the highest cross-validation stability, maintaining top-5 rankings in ≥86% of folds across both RF and XGBoost, while BUN maintained up to 98% stability in RF. For HHS improvement, GPT, cardiovascular comorbidity, BUN, and age anchored feature importance in both models, with GPT and cardiovascular status exhibiting up to 88% top-5 selection frequencies in RF (Supplementary Table S3). For NRS pain relief, DXA T-score, platelet count, serum sodium, and age consistently dominated model predictions across resampling iterations, confirming skeletal, hematologic, and electrolyte status as robust correlates of pain reduction.

#### 3.3.2. SHAP Dependence Analysis and Cutoff Values

**Figure 3:**
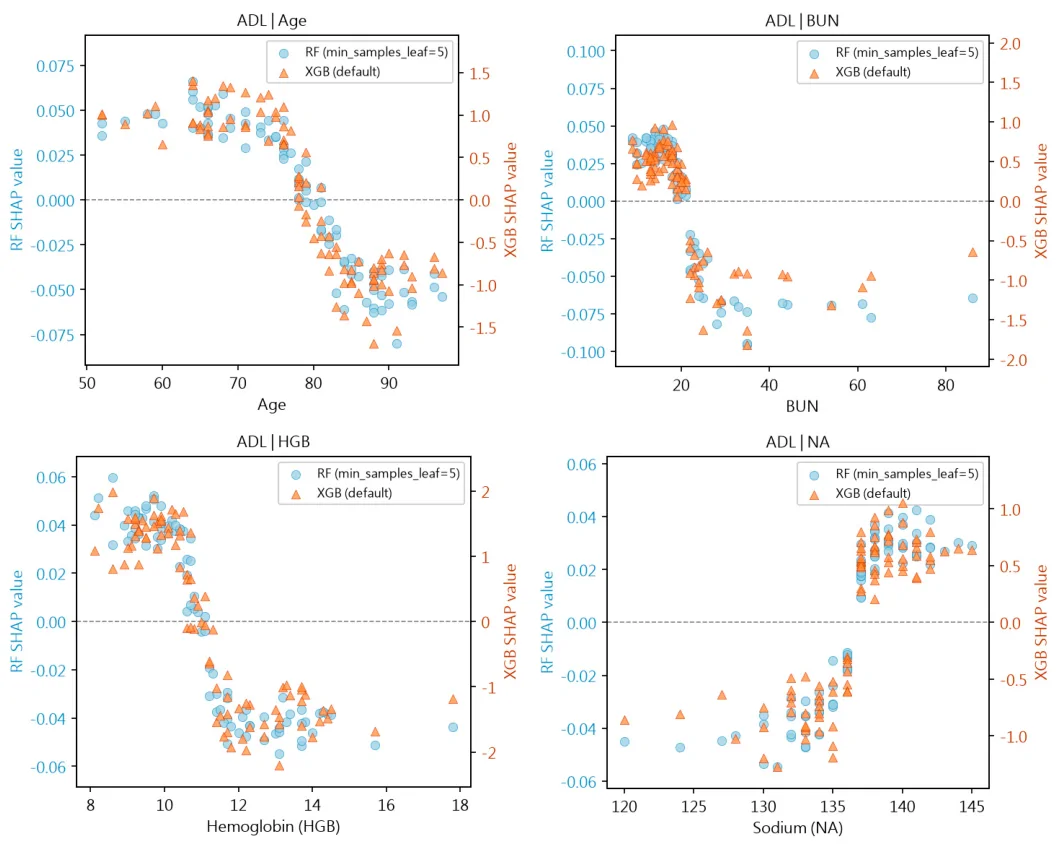
Overlaid out-of-fold SHAP dependence plots for key physiological features associated with ADL improvement. Blue circles indicate Random Forest, orange triangles indicate XGBoost, and each point represents an individual patient. BUN, blood urea nitrogen; HGB, Hemoglobin; NA, Sodium; SHAP, Shapley additive explanations.

**Figure 4:**
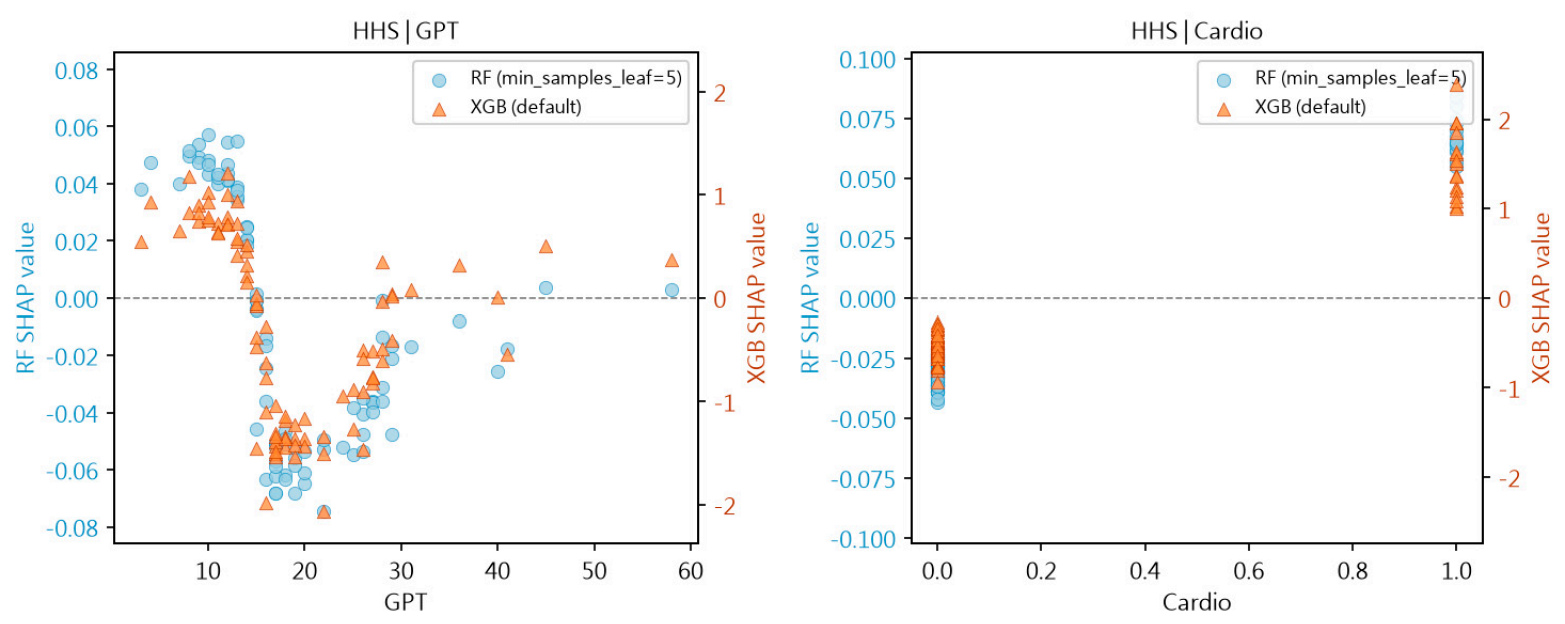
Overlaid out-of-fold SHAP dependence plots for key physiological features associated with HHS improvement. Blue circles indicate Random Forest, orange triangles indicate XGBoost, and each point represents an individual patient. GPT, GPT/ALT; Cardio, Cardiovascular comorbidity; SHAP, Shapley additive explanations.

**Figure 5:**
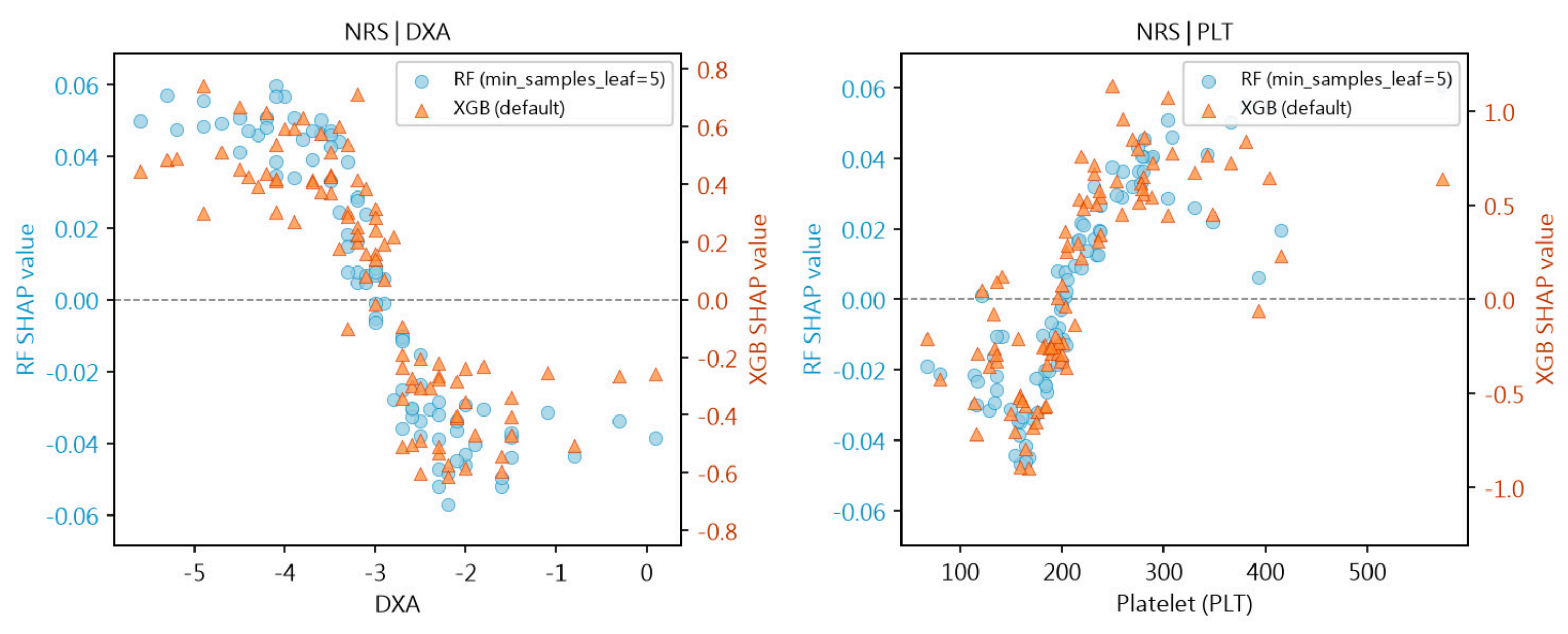
Overlaid out-of-fold SHAP dependence plots for key physiological features associated with pain reduction during inpatient rehabilitation. Blue circles indicate Random Forest, orange triangles indicate XGBoost, and each point represents an individual patient. DXA, Bone mineral density (DXA T-score); PLT, Platelet count; SHAP, Shapley additive explanations.

To characterize how individual continuous and categorical variables shaped model outputs, SHAP dependence analysis was synthesized across both Random Forest and XGBoost architectures. The two models showed highly consistent results, with similar non-linear trajectories and clinical thresholds across the key predictors (Figure 3, Figure 4 and Figure 5).

A. ADL improvement

SHAP analysis identified age, BUN, hemoglobin, and sodium as the main features associated with ADL improvement (Figure 3):

- **Age:** Showed a threshold around 78–80 years. Positive SHAP values were observed below this age, whereas contributions became negative thereafter.
- **BUN:** Showed a threshold around 20 mg/dL. BUN levels below 20 mg/dL were associated with positive SHAP values, whereas higher levels showed negative contributions.
- **Hemoglobin:** Showed a threshold around 11 g/dL. Hemoglobin levels above 11 g/dL were associated with positive SHAP values.
- **Sodium:** Showed a threshold at 135 mEq/L. Sodium levels ≥135 mEq/L were associated with positive SHAP values.

B. HHS Improvement

SHAP analysis identified GPT/ALT and cardiovascular comorbidity as the main features associated with HHS improvement (Figure 4):

- **GPT/ALT:** Showed a clear threshold around 15–20 U/L. Lower baseline GPT/ALT levels were associated with positive SHAP values, whereas higher levels showed predominantly negative contributions.
- **Cardiovascular comorbidity:** Showed a distinct step pattern. Patients with cardiovascular comorbidity (Cardio = 1) had positive SHAP values, whereas those without cardiovascular comorbidity (Cardio = 0) had negative SHAP values.

C. NRS Pain Reduction

SHAP analysis identified DXA T-score and platelet count as the main features associated with pain reduction during inpatient rehabilitation (Figure 5).

- **DXA T-score**: Showed a clear threshold around −3.0. Lower T-scores were associated with positive SHAP values, whereas higher T-scores were associated with predominantly negative contributions.
- **Platelet count**: Showed a threshold around 190–200 × 10^3^/μL. Platelet counts above this level were associated with positive SHAP values, whereas lower counts showed predominantly negative contributions.

## 4. Discussion

### 4.1. Summary of Main Findings and Classification Feasibility

This study’s primary aim was to identify key admission-time clinical predictors and non-linear thresholds associated with functional and pain recovery across daily living (ADL), hip function (HHS), and pain (NRS) outcomes in older adults undergoing subacute inpatient rehabilitation after fragility fracture surgery, with a secondary aim of evaluating the feasibility of explainable ML for outcome classification. Regarding this secondary, feasibility-level aim, all three algorithms (LR, RF, and XGBoost) demonstrated modest baseline discrimination (AUC 0.52–0.67) without significant performance superiority after multiple comparison adjustments. This modest performance is clinically plausible because our feature set deliberately excluded baseline functional scores to avoid circularity with outcome definitions, despite systematic reviews confirming premorbid functional status as a primary prognostic driver [19,20]. As an exploratory study constrained by a modest sample size, these findings should be regarded as hypothesis-generating rather than confirmatory; further validation will be needed before this approach could inform clinical decision-making.

### 4.2. Determinants of ADL Recovery: Hematologic and Metabolic Thresholds

On the factor-identification aim, SHAP analysis revealed a biologically plausible predictor set for ADL improvement, consistently anchored by hemoglobin (HGB), blood urea nitrogen (BUN), serum sodium (Na+), and age. While prior systematic reviews established that advanced age, biochemical derangements, and preoperative anemia correlate with poorer functional gain [19,21,22], our dependence analysis extends the literature by providing quantified clinical inflection thresholds (Figure 3):

**Hemoglobin & Renal Hydration:** Positive SHAP contribution was maintained above 11 g/dL for hemoglobin and below 20 mg/dL for BUN, representing vital physiological thresholds for tissue oxygenation and renal-hydration status. Although some observational reports found no association between discharge hemoglobin and functional recovery [23], our findings align with evidence that admission anemia impairs physical recovery and quality of life [21].

**Serum Sodium & Age:** Normonatremia (≥135 mEq/L) favored ADL recovery, mirroring meta-analytic evidence linking preoperative hyponatremia to increased postoperative complications and frailty [24,25]. For age, negative attribution accelerated past 80 years, corroborating evidence that the advanced elderly population drives age-related recovery deficits [19,20].

### 4.3. Pain Trajectory Dynamics (NRS): Bone Density and Platelet Indices

Classifying NRS pain reduction from admission data alone presented a unique challenge, consistent with literature demonstrating that post-acute pain trajectories are heavily influenced by unmeasured psychosocial factors, active analgesic management, and multidimensional clinical risks [26,27]. Nevertheless, SHAP dependence identified DXA T-score and platelet count as key primary features:

**DXA T-score:** Worse bone mineral density (T-score ≤ −3.0) generated positive SHAP contributions toward pain relief. While osteoporosis is increasingly conceptualized as a marker of systemic frailty [28], this inverse relationship is unexpected and lacks a direct mechanistic explanation. It likely reflects unmeasured confounding (e.g., differences in fracture patterns, surgical approaches, or post-acute analgesic regimens in severe osteoporotic patients) and should be viewed as hypothesis-generating.

**Platelet Count:** Platelets displayed a monotonic positive association crossing zero into favorable attribution at approximately 190–200 × 10^3^/μL. Given limited literature on platelet kinetics and subacute pain resolution, this observation warrants further mechanistic exploration.

### 4.4. Hip Functional Recovery (HHS) and Comorbidity Observations

For HHS improvement, hepatic status (ALT/GPT ≤ 15–20 U/L), renal markers, age, and cardiovascular comorbidity were the most consistent contributors. While age and systemic comorbidity burden are both well-established predictors of hip rehabilitation outcomes [20,29,30], with comorbidity burden showing a moderate inverse pooled correlation with functional status (r = −0.43) in a dedicated meta-analysis, the positive SHAP attribution observed for cardiovascular comorbidity in this small cohort represents a potential clinical paradox. Rather than a genuine protective effect of the disease itself, this non-intuitive positive association between cardiovascular comorbidity and HHS improvement may instead reflect unmeasured pharmacotherapeutic and perioperative monitoring factors. Patients with known cardiovascular conditions are subject to heightened perioperative vigilance and risk-adjusted inpatient protocols [31].

Furthermore, these patients routinely receive long-term cardiovascular management—such as statins (which possess anti-inflammatory properties) or blood pressure optimization. Enhanced peripheral muscle perfusion and attenuated systemic inflammation from such medical optimization may inadvertently support physical engagement and functional mobility during inpatient rehabilitation. This medication-related mechanism, together with closer perioperative monitoring, offers one plausible explanation for the counterintuitive association, though small-sample instability cannot be fully excluded as a contributing factor (see Strengths and limitations).

Finally, when assessing hip functional gains during inpatient rehabilitation, evaluating specific functional sub-domains provides enhanced outcome reliability over crude demographic variables alone [32].

### 4.5. Comparison with Emerging Large Language Models (LLMs)

Recently, large language models (LLMs) have also been explored in fragility-fracture risk prediction. For instance, Alshraideh et al. (2025) [33] utilized transformer-based LLMs (LLaMA-7B and Gemma-7B) to predict recurrent fragility fractures, reporting high performance (AUC 0.92–0.93). Both studies converge on age and bone density (DXA) as key determinants. However, key methodological differences explain the performance disparity:

1. **Model Paradigm**: While generative LLMs excel at complex sequential pattern recognition, our study employed traditional supervised machine learning algorithms (e.g., Random Forest, XGBoost) specifically tailored for tabular clinical feature classification.
2. **Data Integrity vs. Synthetic Oversampling**: Alshraideh et al. applied SMOTE oversampling, which generates synthetic data that can artificially inflate apparent accuracy. In contrast, we strictly preserved the natural clinical distribution without synthetic manipulation to avoid distorting real-world feature relationships.
3. **Endpoint Dynamics**: Predicting long-term fracture recurrence represents a distinct endpoint compared to classifying subacute functional and pain gains during inpatient rehabilitation—an outcome heavily shaped by unmeasured perioperative and rehabilitative variables.

Therefore, direct metric comparison between generative LLMs using synthetic data and supervised ML operating on raw clinical cohorts should be interpreted with caution.

### 4.6. Strengths and Limitations

The present study was strengthened by the use of a single, pre-specified hybrid model configuration applied identically across all three outcomes, repeated stratified cross-validation, and formal statistical comparison between models (corrected resampled *t*-test, DeLong’s test, and McNemar’s test), which together reduce the risk of selectively reporting the best-performing configuration. The addition of a SHAP stability analysis across cross-validation folds further supports the credibility of the identified feature associations. Additionally, the study used real-world, multi-dimensional clinical data, increasing its relevance to routine clinical settings.

Several limitations should be considered when interpreting our findings. First, the single-center retrospective design introduces inherent selection bias and sample constraints (n = 88). Small-sample instability may also contribute to counterintuitive associations such as the DXA T-score finding for NRS, and cannot be fully excluded for the cardiovascular-comorbidity finding despite the alternative medication-related explanation proposed in Section 4.4; formal model calibration was similarly precluded by the sample size. Second, while class weighting preserved the authentic clinical distribution, effective sample sizes for minority classes remained small (23 improved for HHS; 31 unimproved for NRS). Additionally, outcome definitions presented technical trade-offs: although the HHS, ADL, and NRS thresholds were all anchored to external literature (total hip arthroplasty, post-acute geriatric rehabilitation, and chronic pain cohorts, respectively), none were derived or validated specifically within a fragility-hip-fracture rehabilitation population, and future work should test model sensitivity across alternative, population-specific cutoffs. Model-specific instability was also evident for platelet count in the ADL model, which was selected among the top-5 features in only 32% of cross-validation folds for RF versus 90% for XGBoost; this is consistent with reports that variable-importance rankings from tree ensembles become markedly less stable as the ratio of features to sample size increases [34], and that bagging and boosting algorithms can weight correlated predictors differently—random forests tend to repeatedly select the single strongest correlated feature across bootstrap trees, concentrating importance on one variable, whereas gradient-boosted trees sequentially correct residual errors and can redistribute importance onto correlated, complementary features once the dominant predictor’s signal has been exploited. Finally, deliberately excluding baseline functional scores and discharge-time variables prevented data leakage but inherently constrained overall model discrimination. Consequently, all SHAP-identified associations should be viewed as exploratory rather than causal.

## 5. Conclusions

In conclusion, this exploratory study highlights the potential of explainable AI to uncover non-linear associations between clinical features and functional recovery during inpatient rehabilitation after fragility fracture surgery. Although the current predictive performance precludes immediate standalone clinical application, the identification of candidate factors—hematologic and electrolyte markers for ADL, hepatic, cardiovascular, and renal indices for hip-function recovery, and skeletal and hematologic status for pain relief—provides valuable hypothesis-generating insights. Several of these associations (e.g., cardiovascular comorbidity and bone mineral density) were unexpected in direction and should be treated as preliminary signals rather than established relationships. Future studies with larger, multi-center cohorts are warranted to further validate these findings and refine explainable modeling approaches for clinical research applications.

## Figures and Tables

**Table 1:** Baseline characteristics of the study cohort, overall and stratified by rehabilitation improvement status across ADL, HHS, and NRS outcomes.

Variable	All (n = 88)	ADL (BI)		HHS		NRS	
		Improved (n = 46)	Not Improved (n = 42)	Improved (n = 23)	Not Improved (n = 65)	Improved (n = 57)	Not Improved (n = 31)
Gender							
Male	36 (40.9%)	15 (32.6%)	21 (50.0%)	8 (34.8%)	28 (43.1%)	21 (36.8%)	15 (48.4%)
Female	52 (59.1%)	31 (67.4%)	21 (50.0%)	15 (65.2%)	37 (56.9%)	36 (63.2%)	16 (51.6%)
Operation type							
ORIF	44 (50.0%)	21 (45.7%)	23 (54.8%)	9 (39.1%)	35 (53.8%)	30 (52.6%)	14 (45.2%)
Bipolar	44 (50.0%)	25 (54.3%)	19 (45.2%)	14 (60.9%)	30 (46.2%)	27 (47.4%)	17 (54.8%)
Age (years)	77.68 (10.57)	74.89 (10.48)	80.74 (9.91)	76.35 (8.86)	78.15 (11.14)	77.28 (8.89)	78.42 (13.24)
Diabetes mellitus	34 (38.6%)	16 (34.8%)	18 (42.9%)	13 (56.5%)	21 (32.3%)	23 (40.4%)	11 (35.5%)
Hypertension	68 (77.3%)	32 (69.6%)	36 (85.7%)	18 (78.3%)	50 (76.9%)	46 (80.7%)	22 (71.0%)
Cerebrovascular accident	6 (6.8%)	2 (4.3%)	4 (9.5%)	1 (4.3%)	5 (7.7%)	4 (7.0%)	2 (6.5%)
Cancer	4 (4.5%)	2 (4.3%)	2 (4.8%)	1 (4.3%)	3 (4.6%)	1 (1.8%)	3 (9.7%)
Neurological disease	6 (6.8%)	2 (4.3%)	4 (9.5%)	0 (0.0%)	6 (9.2%)	3 (5.3%)	3 (9.7%)
Cardiac disease	16 (18.2%)	10 (21.7%)	6 (14.3%)	10 (43.5%)	6 (9.2%)	11 (19.3%)	5 (16.1%)
Renal disease	4 (4.5%)	3 (6.5%)	1 (2.4%)	3 (13.0%)	1 (1.5%)	4 (7.0%)	0 (0.0%)
Genitourinary disease	3 (3.4%)	2 (4.3%)	1 (2.4%)	0 (0.0%)	3 (4.6%)	2 (3.5%)	1 (3.2%)
Bone mineral density (DXA)	−3.01 (1.07)	−2.94 (0.87)	−3.08 (1.26)	−2.88 (1.09)	−3.05 (1.07)	−3.12 (1.05)	−2.79 (1.10)
Hemoglobin (g/dL)	11.19 (1.86)	11.05 (1.97)	11.35 (1.73)	10.87 (1.82)	11.31 (1.87)	11.20 (1.66)	11.18 (2.20)
RDW (%)	13.52 (1.89)	13.27 (1.27)	13.78 (2.37)	13.89 (1.85)	13.38 (1.89)	13.43 (1.58)	13.67 (2.37)
Platelet (×10^3^/μL)	220.26 (81.55)	221.04 (90.03)	219.40 (72.20)	230.43 (92.65)	216.66 (77.71)	222.23 (71.64)	216.65 (98.40)
GOT/AST (U/L)	29.64 (9.80)	29.28 (8.21)	30.02 (11.38)	30.26 (14.77)	29.42 (7.44)	30.75 (9.83)	27.58 (9.56)
GPT/ALT (U/L)	18.85 (9.08)	18.98 (9.42)	18.71 (8.79)	18.09 (10.44)	19.12 (8.62)	19.42 (10.11)	17.81 (6.81)
BUN (mg/dL)	21.25 (12.31)	17.85 (5.71)	24.98 (16.09)	17.04 (3.72)	22.74 (13.88)	19.04 (7.41)	25.32 (17.63)
Sodium (mEq/L)	136.38 (4.20)	137.11 (4.03)	135.57 (4.28)	136.91 (3.87)	136.18 (4.33)	136.95 (4.01)	135.32 (4.42)
Potassium (mEq/L)	3.82 (0.50)	3.80 (0.51)	3.84 (0.49)	3.71 (0.44)	3.85 (0.51)	3.76 (0.53)	3.92 (0.43)
eGFR (mL/min/1.73 m^2^)	84.50 (37.69)	88.04 (33.95)	80.62 (41.47)	88.84 (32.16)	82.96 (39.58)	88.28 (37.66)	77.54 (37.36)

ADL, activities of daily living; HHS, Harris Hip Score; NRS, numeric rating scale; ORIF, open reduction and internal fixation; GOT, serum glutamic oxaloacetic transaminase; GPT, serum glutamic pyruvic transaminase; BUN, blood urea nitrogen; GFR, estimated glomerular filtration rate. Values are presented as mean (SD) for continuous variables and n (%) for categorical variables. These are descriptive baseline comparisons between subgroups; no formal hypothesis testing was performed given the number of variables shown, to avoid inflating the risk of false-positive (Type I error) findings from multiple univariate comparisons.

**Table 2:** Model performance for ADL, HHS, and NRS improvement by classification metrics.

Outcome	Model	AUC	F1	Sensitivity	Specificity	Balanced Accuracy
ADL	LR	0.671 (0.131)	0.614 (0.121)	0.618 (0.166)	0.591 (0.178)	0.604 (0.114)
	RF	0.601 (0.122)	0.591 (0.107)	0.615 (0.152)	0.516 (0.153)	0.565 (0.092)
	XGB	0.624 (0.114)	0.593 (0.119)	0.611 (0.166)	0.537 (0.178)	0.574 (0.109)
HHS	LR	0.601 (0.148)	0.353 (0.175)	0.442 (0.245)	0.651 (0.136)	0.546 (0.133)
	RF	0.668 (0.148)	0.420 (0.156)	0.462 (0.193)	0.749 (0.125)	0.606 (0.104)
	XGB	0.636 (0.152)	0.310 (0.190)	0.298 (0.186)	0.800 (0.120)	0.549 (0.109)
NRS	LR	0.515 (0.141)	0.597 (0.122)	0.564 (0.153)	0.439 (0.199)	0.502 (0.127)
	RF	0.566 (0.138)	0.671 (0.127)	0.673 (0.180)	0.448 (0.186)	0.560 (0.119)
	XGB	0.530 (0.129)	0.667 (0.093)	0.680 (0.136)	0.376 (0.160)	0.528 (0.098)

Values are presented as mean (SD) derived from 50 splits of repeated stratified 5-fold cross-validation. ADL, activities of daily living; HHS, Harris Hip Score; NRS, numeric rating scale; AUC, area under the receiver operating characteristic curve; LR, logistic regression; RF, random forest; XGB, Extreme Gradient Boosting.

**Table 3:** Out-of-Fold TreeSHAP Feature Importance Ranking by Outcome and Model.

Outcome	Rank	RF’s Feature	Mean |SHAP|	XGB’s Feature	Mean |SHAP|
ADL	1	BUN	0.038	HGB	1.243
	2	Age	0.037	PLT	0.827
	3	HGB	0.037	Age	0.835
	4	NA	0.030	NA	0.669
	5	PLT	0.019	BUN	0.655
HHS	1	GPT	0.040	GPT	0.837
	2	Cardio	0.038	Age	0.826
	3	BUN	0.034	Cardio	0.723
	4	Age	0.033	BUN	0.563
	5	HGB	0.025	GOT	0.513
NRS	1	DXA	0.034	NA	0.564
	2	PLT	0.025	HGB	0.480
	3	NA	0.024	PLT	0.474
	4	Age	0.021	Age	0.451
	5	K	0.020	GOT	0.423

Note: ADL, Activities of Daily Living; BUN, Blood Urea Nitrogen; Cardio, Cardiovascular Comorbidity; DXA, Bone Mineral Density (DXA T-score); GOT, Aspartate Aminotransferase (AST); GPT, Alanine Aminotransferase (ALT); HGB, Hemoglobin; HHS, Harris Hip Score; K, Serum Potassium; NA, Serum Sodium; NRS, Numeric Rating Scale; PLT, Platelet Count; RF, Random Forest (min_samples_leaf = 5); SHAP, Shapley Additive Explanations; XGB, XGBoost. Values represent mean absolute out-of-fold SHAP values ($\mathrm{mean}\left(\left|\mathrm{SHAP}\;\mathrm{value}\right|\right)$) calculated across 50 repeated cross-validation folds, where higher values denote greater overall predictive influence. Within each model, features are ranked by mean rank across the 50 cross-validation folds rather than by this single-point Mean |SHAP| estimate, which better reflects selection stability and can occasionally reorder features with closely similar mean |SHAP| magnitudes (e.g., platelet count vs. age for ADL in the XGBoost model). Feature rank stability across cross-validation folds was audited separately (see Supplementary Table S3 for rank means, standard deviations, and selection frequencies).

## Data Availability

The datasets used and/or analysed during the current study are available from the corresponding author on reasonable request.

## Supplementary Materials

The following supporting information can be downloaded at: https://www.mdpi.com/article/10.3390/diagnostics16172875/s1. Supplementary Table S1: Definition of Comorbidity Categories by ICD-10 Codes. Supplementary Table S2: Sensitivity analysis comparing default versus regularized algorithm configurations for ADL, HHS, and NRS improvement. Supplementary Table S3: Feature Rank Stability and Top-5 Selection Frequency Across 50 Repeated Cross-Validation Folds.
