# Supplementary material for: Explainable Machine Learning Models for Predicting Functional and Pain Recovery After Fragility Fracture Surgery
Source: Diagnostics (Basel). 2026 Sep 7;16(17):2875. doi: 10.3390/diagnostics16172875 (PMC13565305; doi:10.3390/diagnostics16172875)
Supplement: Supplementary file 1 [file diagnostics-16-02875-s001.zip › diagnostics-4375795-supplementary.pdf]

Supplementary Table S1. Definition of Comorbidity Categories by ICD-10 Codes

COMORBIDITY CATEGORY	DEFINITION	INCLUDED CONDITIONS	ICD-10 CODES
**CANCER**	Malignant neoplasms	Solid and hematologic malignancies	C00–C97
**NEUROLOGICAL DISEASE**	Disorders of the nervous system	Stroke, dementia, Parkinson’s disease, epilepsy	I60–I69, G20, G30, G40
**CARDIOVASCULAR DISEASE**	Diseases of the circulatory system	Hypertension, ischemic heart disease, heart failure, arrhythmia	I10–I15, I20–I25, I50, I47–I49
**RENAL DISEASE**	Chronic kidney disorders	Chronic kidney disease, renal failure	N18, N19
**GENITOURINARY DISEASE**	Disorders of the genitourinary system	Benign prostatic hyperplasia, urinary tract disorders	N40, N30–N39
**LIVER / ENDOCRINE DISEASE**	Hepatic and endocrine disorders	Chronic liver disease, diabetes mellitus, thyroid disorders	K70–K77, E10–E14, E00–E07

ICD-10, International Classification of Diseases, 10th Revision

Supplementary Table S2. Sensitivity analysis comparing default versus regularized algorithm configurations for ADL, HHS, and NRS improvement.

Model	Outcome	Variant (Configuration)	AUC	Specificity			Balanced Accuracy		
			Mean(SD)	Mean(SD)	t_corrected†	p_corrected	Mean(SD)	t_corrected†	p_corrected
**LR**	ADL	Default	0.671(0.131)	0.591(0.178)	-0.011	0.992	0.604(0.114)	-0.233	0.817
		Regularized (C=0.1)	0.693(0.133)	0.591(0.182)			0.614(0.110)		
	HHS	Default	0.601(0.148)	0.651(0.136)	1.675	0.100	0.546(0.133)	0.344	0.732
		Regularized (C=0.1)	0.562(0.150)	0.582(0.148)			0.522(0.136)		
	NRS	Default	0.515(0.141)	0.439(0.199)	-0.562	0.576	0.502(0.127)	-0.726	0.471
		Regularized (C=0.1)	0.549(0.142)	0.488(0.193)			0.538(0.110)		
**RF**	ADL	Default	0.599(0.124)	0.480(0.170)	-0.457	0.650	0.559(0.097)	-0.134	0.894
		Regularized (min_samples_leaf=5)	0.601(0.122)	0.516(0.153)			0.565(0.092)		
	HHS	Default	0.651(0.153)	0.797(0.119)	1.051	0.299	0.580(0.111)	-0.555	0.582
		Regularized (min_samples_leaf=5)	0.668(0.148)	0.749(0.125)			0.606(0.104)		
	NRS	Default	0.587(0.129)	0.377(0.179)	-0.968	0.338	0.561(0.100)	0.008	0.994
		Regularized (min_samples_leaf=5)	0.566(0.138)	0.448(0.186)			0.560(0.119)		
**XGB**	ADL	Default	0.624(0.114)	0.537(0.178)	0.017	0.986	0.574(0.109)	-0.144	0.886
		Regularized (min_child_weight=5)	0.635(0.123)	0.536(0.155)			0.582(0.103)		
	HHS	Default	0.636(0.152)	0.800(0.120)	1.355	0.182	0.549(0.109)	0.102	0.919

		Regularized (min_child_weight=5)	0.610(0.135)	0.722(0.120)			0.544(0.111)		
		Default	0.530(0.129)	0.376(0.160)	-0.808	0.423	0.528(0.098)	-0.180	0.858
	NRS	Regularized (min_child_weight=5)	0.536(0.134)	0.458(0.215)			0.539(0.106)		

† *$t_{corrected}$ values were derived from Nadeau and Bengio's corrected resampled t-test calculated as $(Mean_{default} - Mean_{regularized})$; positive t-values indicate higher metric performance in the default configuration, whereas negative values indicate higher performance in the regularized configuration.*

Supplementary Table S3. Feature Rank Stability and Top-5 Selection Frequency Across 50 Repeated Cross-Validation Folds.

Outcome	Rank	RF's Feature	Rank Mean(SD)	Top-5 selection frequency (%)	XGB's Feature	Rank Mean(SD)	Top-5 selection frequency (%)
**ADL**	**1**	**Age**	1.64(1.80)	94%	**HGB**	0.44(0.85)	100%
	**2**	**BUN**	1.66(1.39)	98%	**PLT**	2.32(1.59)	90%
	**3**	**HGB**	1.72(1.58)	96%	**Age**	2.40(1.97)	86%
	**4**	**NA**	3.18(2.46)	78%	**NA**	3.86(2.68)	72%
	**5**	**PLT**	6.04(3.28)	32%	**BUN**	4.04(2.65)	64%
**HHS**	**1**	**GPT**	1.82(1.74)	88%	**Age**	2.24(2.69)	82%
	**2**	**Cardio**	2.24(1.88)	86%	**GPT**	2.34(2.49)	82%
	**3**	**BUN**	2.44(1.99)	82%	**Cardio**	3.14(2.65)	72%
	**4**	**Age**	2.50(1.94)	86%	**BUN**	4.48(2.86)	54%
	**5**	**GOT**	4.96(2.16)	40%	**GOT**	4.50(2.87)	52%
**NRS**	**1**	**DXA**	1.98(2.44)	84%	**NA**	3.72(3.04)	60%
	**2**	**PLT**	3.06(2.44)	78%	**Age**	3.92(2.91)	64%
	**3**	**Age**	3.38(2.31)	70%	**PLT**	3.94(3.15)	60%
	**4**	**NA**	4.14(3.16)	60%	**HGB**	4.30(2.84)	56%
	**5**	**K**	4.92(3.17)	40%	**GOT**	4.78(3.19)	50%

Note: ADL, Activities of Daily Living; HHS, Harris Hip Score; NRS, Numeric Rating Scale; RF, Random Forest; SD, Standard Deviation; XGB, XGBoost. Feature rank stability was evaluated across 50 repeated cross-validation folds. Rank, Mean (SD) represents the average feature importance rank and its standard deviation across folds, where a lower mean rank indicates greater baseline predictive influence. Top-5 selection frequency (%) indicates the percentage of cross-validation folds in which the feature ranked among the top 5 most influential predictors.
